# On Two Cases of Hysteria

**Published:** 1897-12-18

**Authors:** Dyce Duckworth

**Affiliations:** Physician to the Hospital


### Medical Progress and Hospital Clinics.

[*The Editor will be glad to receive offers of co-operation and contributions from members of the profession. All letters should be addressed to* The Editor, at the Office, 28 & 29, Southampton Street, Strand, London, W.C.]

### ON TWO CASES OF HYSTERIA.

Abstract of a clinical lecture given at St. Bartholomew's Hospital by Sir Dyce Duckworth, M.D., F.R.C.P., Physician to the Hospital.

(*Reported for* The Hospital, *and corrected by the Author.*)

The first case is that of a married woman, without family, age 28, who was admitted into the hospital on October 23rd, 1897. The history is that she has been the subject of fits, *i.e.*, convulsive attacks, since an early age. The first fit occurred when she was seven years old. During the ensuing three years she had no fits, when they again commenced, and have continued ever since. During the last four months the intervals between the fits have decreased, so that she now has two or three per day. Any excitement brings on a fit (the onset of which is sudden), there being no warning except a peculiar feeling over the body. She has been married ten years, has not had any children, and has never had any miscarriages. The menstruation has always been regular. The family history, as far as can be learned, is negative, none of her relatives having had nervous disease or fits. She is supposed to suffer from epilepsy. The patient is a stout, dark, healthy-looking woman. On examination, there are no physical signs, excepting that there is slight tenderness on pressure in the left iliac region. The pulse is a little irregular, the lungs are normal, the knee-jerks are natural, and there is no ankle-clonus. When the fits come on, she sits up in bed, with a terrified expression, and calls for assistance; and then movements of the arms and legs commence. She does not know what happens during the attack. She has often fallen down during the fits, and has, it is said, bitten her tongue, but she has never hurt herself. So that this is a case of nervous disease and convulsive attacks without apparent cause.

The second case is a single women, aged 22, a domestic servant. She was in the hospital for several months some time ago, and was discharged cured. She then went home for six months, and has now been readmitted as bad as ever. The history is that two years before admission she had a serious fall, but her friends thought that previous to this she had dragged her right leg a little in walking. She has had sensations of "pins and needles" in the limbs, and is said to have had fits when 14 years of age. The menstruation has been normal. The family history is that her mother was paralysed, and that her paternal aunt had fits. It was noticed that when this patient was sitting there was no tremor or disquietude of the limbs. She was then made to walk across the floor of the lecture theatre, and when doing so it was noticed that she had tremors of all the limbs, and that she brought the feet round one in front of the other, rolled from side to side, and appeared to be on the point of falling down, though she did not fall, and she rarely has fallen. She was sent into hospital supposed to be suffering from a cerebral tumor. Repeated examinations of the fundus of the eye have shown the retinæ to have been normal. The temperature has always been slightly subnormal.

Regarding these two cases, Sir Dyce Duckworth remarked that they were examples of a condition which was not uncommon, being, in fact, two cases of hysteria. Hysteria, he said, is often difficult to diagnosticate, for it assumes so many forms and simulates every disease. If every part of the body were examined microscopically it would probably be found that this disease has no gross morbid anatomy; so one must think of it as a "state" or "peculiar mode of working," of the nervous system. The subjects of it are usually females, though one occasionally sees cases of hysteria in youths and men. In women it occurs between the ages of 15 and 40; that is to say, during the period that the uterine functions are active. It is not necessarily connected with disease of the genital organs, but disease of the genital organs may aggravate it.

It has been said that if careful and complete observation were made it would be found that every woman is in some degree hysterical. Within the last 50 years, however, hysteria appears to have become less common, for if one reads the novels, like those of Dickens and Thackeray, which were written 50 years ago, one finds that most of the female characters described in those books were prone to go off into hysterical manifestations on slight provocation. Formerly, living in close rooms, taking no exercise, and wearing tight stays aggravated or encouraged this tendency. But nowadays these conditions are greatly altered, for women lead more active lives, and compete with men in many walks of life. Yet some women inherit an unstable nervous system. Sir James Paget's name for this disorder is "neuromimesis," that is, nerve-mimicry. It is not malingering, for the nerves are working wrongly, and the patients are really ill. It imitates other diseases; but this it does unconsciously, for cases of hysteria simulating other diseases have occurred in girls who have lived in remote parts of the country, and have never seen the nervous maladies they simulated. There is no disease, even the most rare, which has not been imitated by hysteria. Cancer, strumous conditions, and all kinds of nervous diseases have been simulated by it. Now the question arises: Given this peculiar "mode of working" of the nervous system, what is the exciting cause of the manifestations of the disease? There are, in truth, many determining causes. It may be a shock, such as a fall, or a great emotion, such as a death in the family, disappointment in love, which is apt to be more acutely felt by women than by men, a fright, long-continued annoyance at home, chagrin. And it is necessary for a doctor who wishes to understand these cases thoroughly to consider fully all these possible causes of the disease.

The remedy is to do as was done in this hospital with the above case—that is to say, she was drilled, exercised, and ordered about and made to follow some occupation. A little ridicule, too, was found beneficial. The result was that she got quite well, She then returned to her home, and gradually her symptoms came back, even more markedly than before.

These cases illustrate the two varieties that are met with in hysteria, namely: (1) The convulsive, and (2) the non-convulsive. It is to be remembered that it is the will-power which is at fault. These patients have insufficient power to control or will to do anything. Amongst cases like the two above described we meet with the celebrated "fasting girls." There was one in Wales in 1866, and skilled nurses were sent down to her from a London hospital to see if the fasting was real. It was found that she did fast, and she was allowed to die. Nowadays a case like this is forcibly fed by a nasal tube and recovers. These patients sometimes eat all kinds of extraordinary things, such as slate pencils, candles, and even fæces. They try to deceive you, and will bring you a pot of blood that they pretend to have vomited, or they will swallow blood and vomit it, in order to excite sympathy—for all their performances take place in public. The convulsive case which has been shown will be douched with cold water, kept warm and comfortable, well fed, put under strict discipline, and isolated, and she will not be allowed to write letters to her friends or to receive letters from them. Massage will be employed, that is to say, the Weir-Mitchell treatment will be carried out. Home is the worst place for them. Marriage does not always cure them, and nothing is worse than the fond parent or husband. "The velvet glove on the iron hand" is the treatment for these cases. It is sometimes good to give the so-called stinking drugs, such as asafœtida. Beware of making frequent vaginal examinations, and do not pay too much attention to the uterus, or to any other organ, for as surely as you begin to do so, it will begin to ache. Do not give narcotic drugs, such as opium, to produce sleep, or you will find that your patient cannot do without them, and that she requires more and more of them. Do not give stimulants, or these patients may become drunkards. There is nothing so good as cold water. If the patient is in a fit get a jug of cold water, do not heed her remonstrances, but holding it well above the patient pour on. Some of these patients cannot swallow, then pass a big bougie, and before it gets half way down the œsophagus they will have recovered. Some of these patients cannot speak. Then get a battery, place one pole over the thyroid cartilage and the other at the nape of the neck, and give them a strong shock; they will promptly scream, so that they can be heard outside the ward. Some of these cases are very puzzling ones, because hysteria takes so many different forms that one may easily be deceived.

Regarding the pathology of this disease, it verges on insanity, and some of these unfortunate patients do become insane and end their days in an asylum. In the present state of our knowledge hysteria must be regarded as a crazy way of working of the nervous system. It is hereditary. The moral, therefore, is, said the lecturer, to grow up a strong, healthy set of men and women, and I think that now, in the closing years of the nineteenth century, we are on our way to do it.

